# Site- and allele-specific polycomb dysregulation in T-cell leukaemia

**DOI:** 10.1038/ncomms7094

**Published:** 2015-01-23

**Authors:** Jean-Marc Navarro, Aurore Touzart, Lydie C. Pradel, Marie Loosveld, Myriam Koubi, Romain Fenouil, Sandrine Le Noir, Muhammad Ahmad Maqbool, Ester Morgado, Claude Gregoire, Sebastien Jaeger, Emilie Mamessier, Charles Pignon, Salima Hacein-Bey-Abina, Bernard Malissen, Marta Gut, Ivo G. Gut, Hervé Dombret, Elizabeth A. Macintyre, Steven J. Howe, H. Bobby Gaspar, Adrian J. Thrasher, Norbert Ifrah, Dominique Payet-Bornet, Estelle Duprez, Jean-Christophe Andrau, Vahid Asnafi, Bertrand Nadel

**Affiliations:** 1 Center of Immunology of Marseille Luminy, Aix-Marseille University, Parc Scientifique de Luminy case 906, 13288 Marseille, France; 2 INSERM U1104, 13288 Marseille, France; 3 CNRS UMR7280, 13288 Marseille, France; 4 Université Paris Descartes Sorbonne Cité, Institut Necker-Enfants Malades (INEM), Institut national de recherche médicale (INSERM) U1151, and Laboratory of Onco-Hematology, Assistance Publique-Hôpitaux de Paris (AP-HP), Hôpital Necker Enfants-Malades, 75015 Paris, France; 5 Laboratoire Hématologie, APHM, 13385 Marseille, France; 6 CRCM Inserm U1068, Institut Paoli Calmettes; Aix-Marseille Université, UM 105; CNRS UMR7258, 13009 Marseille, France; 7 Biotherapy Department, INSERM U429, Hôpital Necker-Enfants-Malades, 149 rue de Sèvres, 75015 Paris, France; 8 Centro Nacional de Análisis Genómico, 08028 Barcelona, Spain; 9 Department of Hematology, AP-HP Hôpital St-Louis, 75010 Paris, France; 10 Centre for Immunodeficiency, Molecular and Cellular Immunology, Institute of Child Health, University College London, London WC1N 1EH, UK; 11 Services des Maladies du sang CHU and UMR Inserm U 892/CNRS 6299, 49933 Angers, France

## Abstract

T-cell acute lymphoblastic leukaemias (T-ALL) are aggressive malignant proliferations characterized by high relapse rates and great genetic heterogeneity. *TAL1* is amongst the most frequently deregulated oncogenes. Yet, over half of the TAL1^+^ cases lack *TAL1* lesions, suggesting unrecognized (epi)genetic deregulation mechanisms. Here we show that *TAL1* is normally silenced in the T-cell lineage, and that the polycomb H3K27me3-repressive mark is focally diminished in TAL1^+^ T-ALLs. Sequencing reveals that >20% of monoallelic TAL1^+^ patients without previously known alterations display microinsertions or RAG1/2-mediated episomal reintegration in a single site 5′ to *TAL1*. Using ‘allelic-ChIP’ and CrispR assays, we demonstrate that such insertions induce a selective switch from H3K27me3 to H3K27ac at the inserted but not the germline allele. We also show that, despite a considerable mechanistic diversity, the mode of oncogenic *TAL1* activation, rather than expression levels, impact on clinical outcome. Altogether, these studies establish site-specific epigenetic desilencing as a mechanism of oncogenic activation.

## Abstract (web summary)

*TAL1* is frequently deregulated in T-cell acute lymphoblastic leukaemias, but the mechanism remains largely unclear. Here the authors show that microinsertions upstream of *TAL1* cause its epigenetic reactivation, and that the mode of *TAL1* activation correlates with prognosis.

## Graphical abstract

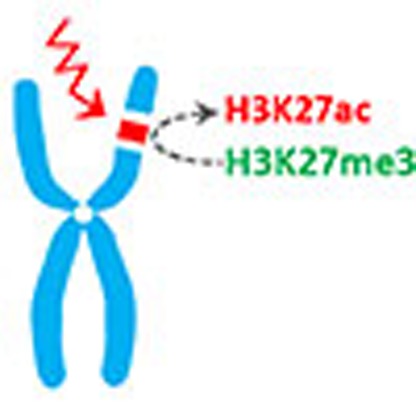

T-cell acute lymphoblastic leukaemia’s (T-ALL) are malignant proliferations of immature T-cell progenitors. Although the outcome of T-ALLs has greatly improved in the last 10 years, ~30% of cases relapse within the first 2 years following diagnosis; moreover, acute- and long-term toxicities remain important issues for long-term survivors, underlining the critical need of better risk stratification of T-ALL patients, and the implementation of more adapted and/or targeted therapies. A major obstacle in the molecular dissection of these processes is that T-ALLs constitute a particularly heterogeneous group of disease, characterized by complex combinations of multigenic aberrations and oncogenic cooperation. The deregulation of over 40 distinct oncogenes and tumour suppressors has been reported, occurring through a large diversity of genomic aberrations and epigenetic mechanisms^1^,^2^. Chromosomal alterations mostly consist of translocations, inversions and microdeletions occurring at the vicinity of proto-oncogenes and leading to their deregulated expression.

*TAL1* is one of the most frequently deregulated T-ALL oncogenes^3^. In physiological conditions, *TAL1* is a regulatory gene that promotes access to alternative fates in haematopoiesis. Expressed in early haematopoiesis, its expression is maintained in the erythroid lineage, but normally irreversibly epigenetically silenced in the T-cell lineage^4^,^5^,^6^ (Supplementary Fig. 1). Events leading to the illegitimate maintenance or re-expression of *TAL1* in the T-cell lineage are thought to constitute strong drivers of T-ALL leukemogenesis. Known *TAL1* dysregulation mechanisms consist of t(1;14)(p34;q11) translocations (1–2%) and SIL-TAL deletions (15–20%). Yet, over half of the TAL1^+^ cases lack *TAL1* lesions, suggesting unrecognized (epi)genetic deregulation pathways^7^. In such ‘unresolved cases’, *TAL1* expression can be monoallelic, compatible with a direct alteration in *cis* within or around the *TAL1* gene, or biallelic, likely reflecting indirect deregulation in *trans*^7^.

Here we sought to gain insights into the (epi)genetic deregulation pathways of *TAL1* ‘unresolved’ cases. Using ChIP-seq and an ‘allelic-ChIP’ assay allowing to discriminate histone marks separately on each allele, we describe a new process of oncogene activation, whereby the targeted eviction of polycomb H3K27me3 marks and concurrent recruitment of H3K27ac marks by micro- and macroinsertional events, trigger the monoallelic desilencing of *TAL1*. Incidentally, we also report the first example of oncogenic activation by recombination-activating gene (RAG)-mediated episomal reinsertion, a very elusive event predicted *in vitro* as a source of oncogenic activation over 10 years ago^8^,^9^, but never identified before in human cancer. We further show that this new epigenetic desilencing process is a recurrent event in T-ALL, accounting for >20% of unexplained cases of monoallelic *TAL1* oncogene activation. Finally, we demonstrate that the mode of activation (monoallelic in *cis* versus biallelic in *trans*) rather than the level of *TAL1* expression impacts on prognosis, with *cis-*mediated alterations significantly associated with adverse clinical outcome.

## Results

### Microinsertions induce epigenetic switch and *TAL1* expression

**Figure 1:**
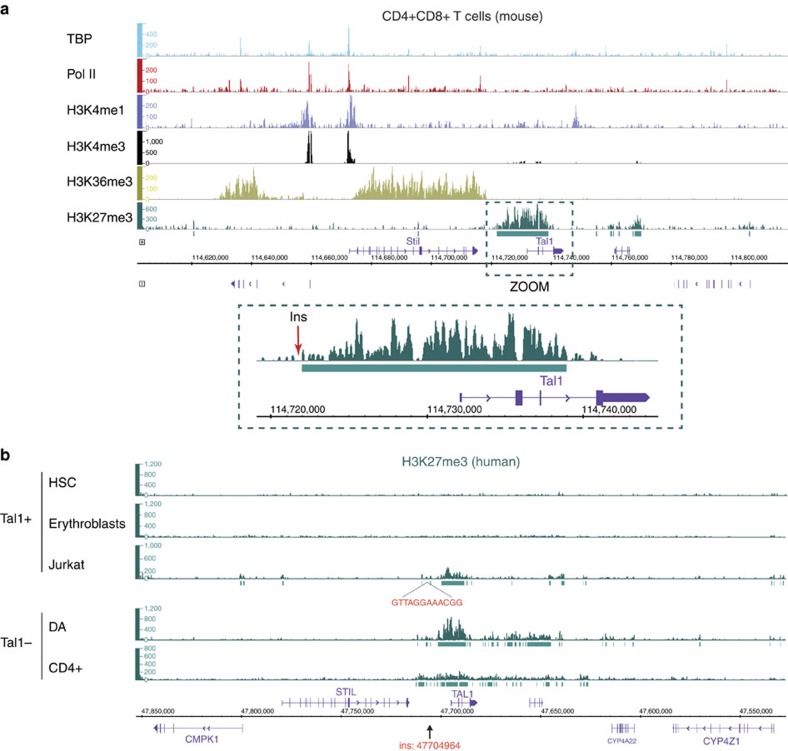
ChIP-seq analysis of the *TAL1* locus in *TAL1*-expressing and *TAL1*-repressed cells. (**a**) ChIP-seq profiles of chromatin marks in normal mouse CD4^+^CD8^+^ thymocytes. The *TAL1* genomic area on chromosome 4 (including the adjacent STIL gene, which is physiologically expressed at that developmental stage) is shown for TATA-binding protein-GTF recruited to promoters (TBP), Polymerase II (Pol II), H3K4me1 (active chromatin mark for enhancer/regulatory regions), H3K4me3 (active chromatin marks for promoters), H3K36me3 (active chromatin marks for gene bodies) and H3K27me3 (repressive chromatin mark dependent on the PcG). The *TAL1* region is zoomed, and the insertion breakpoint localization to the human orthologue region (114,722,607) is indicated by a red arrow. (**b**) ChIP-seq profiles of polycomb repressive chromatin mark H3K27me3 in normal human TAL1^+^ (HSC; erythroblasts) and TAL1^−^ (peripheral CD4^+^ T cells) lineages, compared with TAL1^+^ (Jurkat) and TAL1^−^ (DA) T-ALL cells. The *TAL1* gene and surrounding area on chromosome 1 (including the STIL gene) is shown. The insertion breakpoint localization (47704964, HG19 coordinates) is indicated by an arrow. All profiles were input subtracted, except for HSC and erythroblasts for which input data were not available^12^. Significantly enriched areas are represented as green rectangles under the lanes (MACS2 peaks).

**Figure 2:**
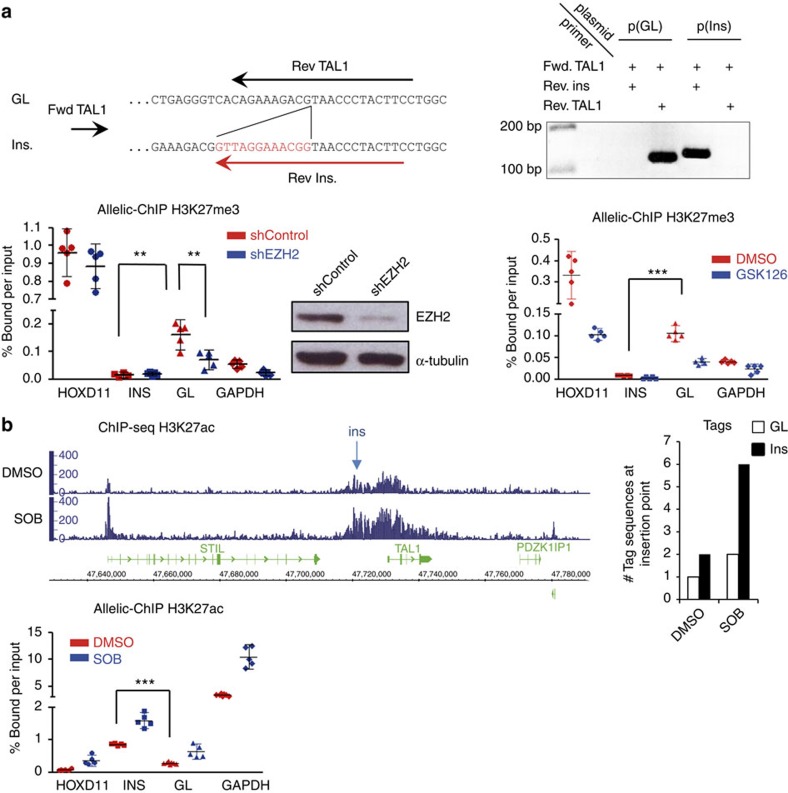
Site- and allele-specific analysis of histone methylation/acetylation marks at the insertion breakpoint in Jurkat. (**a**) Allelic-ChIP assay of H3K27me3 marks. Top panel: the assay to discriminate the germline (GL) from the inserted allele (Ins.) by substituting one of the GL primers allowing detection of the GL configuration at the insertion site (Rev. TAL1), with an insertion-specific primer allowing detection of the inserted configuration (Rev. Ins, overlapping the 12-bp insertion). Primer pairs were tested on GL p(GL) or inserted p(Ins) cloned fragments (and on cell lines containing (Jurkat) or not (DND) the insertion) to exclusively amplify each configuration, and do not crossreact. Bottom middle panel: western blot of EZH2 protein content on shMock or EZH2 knockdown conditions. Allelic-ChIP assays were performed in presence of a non-silencing sh-RNA (shControl) or a sh-RNA-targeting EZH2 (shEZH2) (left panel) or after the incubation of Jurkat cells with GSK126 (0.5 μM, 72H) or vehicle (dimethyl sulphoxide, DMSO; right panel). GAPDH and HoxD11 were used as controls for activated/repressed genes, modulated according to the polycomb-dependent H3K27me3 marks. Note that EZH2 knockdown/inhibition triggered only partial decrease of H3K27me3 marks at the PcG-repressed HoxD11 control gene, possibly due to incomplete knockdown/inhibition and/or redundancy of polycomb components in the adult lymphoid lineage^42^. (**b**) Enrichment of acetylation marks at the *TAL1* locus. H3K27Ac ChIP was performed with Jurkat cells incubated with vehicle (DMSO) or the histone deacetylase inhibitor sodium butyrate (SOB) (5 mM, 4H); DNA was then analysed by ChIP-seq (left panel) or by allelic-ChIP (bottom panel). For the ChIP-Seq, quantification of the number of tag sequences at the insertion point is shown (right panel). ***P<0.001; **P<0.001; *P<0.05, unpaired *t*-test. Errors bars represent 95% confidence interval.

To investigate the (epi)genetic deregulation pathways of TAL1 ‘unresolved’ cases, we first took advantage of chromatin immunoprecipitation coupled to a high-throughput sequencing (ChIP-seq) data set describing active/inactive epigenetic marks in mouse developing thymocytes^6^,^10^. A large region starting upstream of the *TAL1* promoter and encompassing part of the gene body was enriched in H3K27me3, suggesting the involvement of polycomb complex (PcG) repressive activity in *TAL1* silencing (Fig. 1a). This profile was also observed in human TAL1^−^ peripheral CD4^+^ T cells^11^, but absent in TAL1-expressing cells^12^ (CD34^+^ haematopoietic stem cells, HSC; erythroblasts; Fig. 1b), in line with the gradual deposition of H3K27me3-repressive marks during T-cell lineage specification^6^ (Supplementary Fig. 1). To determine if deregulation of this silencing occurs in TAL1^+^ T-ALLs, ChIP-seq was also performed in the Jurkat (TAL1^+^) T-ALL cell-line and a control TAL1^−^ sample (DA). Sample DA displayed the expected H3K27me3 deposition, coherent with *TAL1* silencing in T cells (Fig. 1b). Surprisingly, however, low levels of H3K27me3 marks were also observed in Jurkat. Since, in contrast to normal *TAL1*-expressing cells, Jurkat displays monoallelic expression of TAL1 (Supplementary Fig. 2), we reasoned that a monoallelic chromosomal alteration might have prevented H3K27me3 deposition on one of the two alleles, resulting in an averaged, intermediate ChIP-seq signal. To test this possibility, we sequenced ~10 kb upstream of *TAL1* (Supplementary Fig. 3), covering the regulatory region enriched in H3K27me3 marks. Amid single-nucleotide polymorphisms (SNPs), we found a monoallelic alteration consisting of a 12-bp microinsertion ~7 kb upstream of the *TAL1* p1a promoter, in a region prone to DNA looping with *TAL1* promoters^13^. Interestingly, this insertion mapped at the border of the repressive H3K27me3 pattern (Fig. 1a,b), in line with the possibility that it disrupted normal epigenetic silencing of the *TAL1* locus. To further investigate the association of the insertion with potential allelic distortion of the repressive H3K27me3 mark, we performed an ‘allelic-ChIP’ that discriminates H3K27me3 marks at the inserted versus germline allele (Fig. 2a). A significant decrease of H3K27me3 signal was observed at the inserted allele compared with the germline allele (*P*<0.005). Accordingly, knockdown (by shEZH2) or inhibition (using the EZH2 inhibitor GSK126) (ref. 14) of the Polycomb H3K27me3 methyl transferase EZH2, allowed partial reversion of H3K27me3 deposition at the germline (non-inserted) allele. Considering the cooperative role of histone modifications in expression regulation, we further analysed the relevance of acetylation in presence or absence of histone deacetylase inhibitor (sodium butyrate)^15^. ChIP-seq showed a slight enrichment of H3K27ac with significant overall increase on sodium butyrate treatment (Fig. 2b). Similar to H3K27me3, H3K27ac marks extended to the insertion site, suggesting dual epigenetic regulation and differential allelic recruitment. Indeed, allelic quantification through tag retrieval and allelic-ChIP revealed a difference of H3K27ac between inserted and GL alleles in a pattern symmetrical to and functionally coherent with H3K27me3. Acetylation levels were histone deacetylase inhibitor dependent. Overall, this suggested that Jurkat’s microinsertion contributed to site- and allele-specific switch from H3K27me3 to H3K27ac deposition, leading to the maintenance of *TAL1* expression through T-cell differentiation.

### A recurrent epigenetic mechanism of *TAL1* deregulation

**Figure 3:**
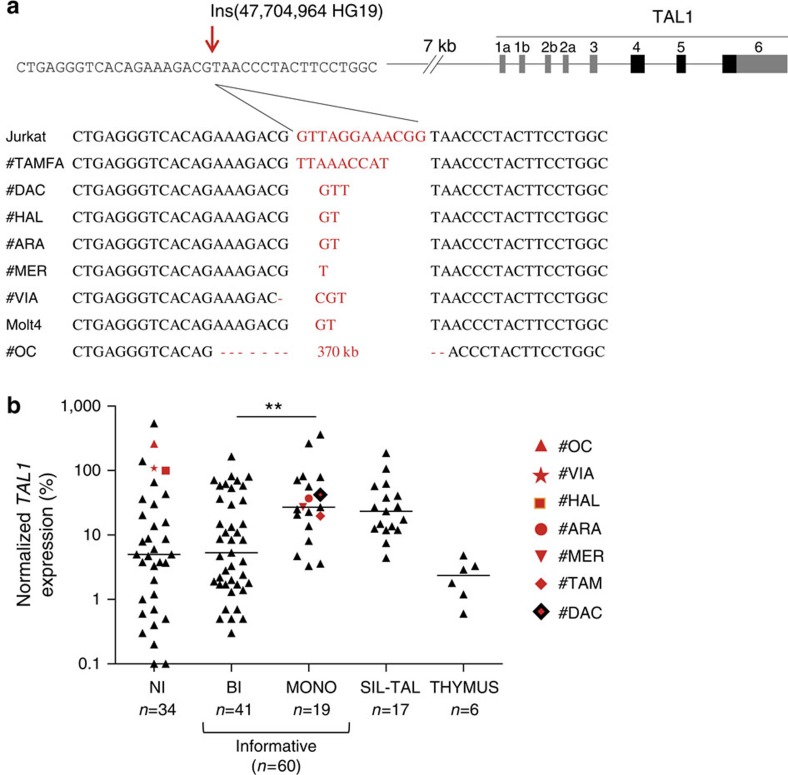
Micro- and episomal insertions are recurrently found in monoallelic TAL1^+^ ‘unresolved cases’. (**a**) Nucleotide sequences of episomal/microinsertions. All insertions were specifically and exclusively located at the indicated genomic position, and are pictured in red. Nucleotide deletions are indicated by a red dash. No SNPs are referenced at this position (Supplementary Fig. 8); (**b**) Relative *TAL1* expression in T-ALL patients (*n*=111) according to biallelic (BI), or monoallelic (MONO) expression; patients with micro/episomal insertions are indicated in red; SIL-TAL cases are shown separately; informative cases: the presence of SNPs in *TAL1* 3’UTR allows to determine if the expression is mono- or biallelic. NI: non-informative cases (absence of SNPs in *TAL1* 3′ UTR does not allow to determine if the expression is mono- or biallelic). The average physiological *TAL1* levels in thymus is shown as reference (Thymus); Horizontal bars indicate median expression levels; **indicates significant difference between BI and MONO expression (Mann–Whitney *U*-test, *P*<0,01); note that a number of biallelic patients are reaching/below physiological thymus levels, and might result from the presence of residual *TAL1*-expressing erythroblasts among tumoral cells^43^; *TAL1* expression was analyzed by Taqman assay and is normalized to ABL (see Methods).

To determine if similar structural abnormalities occurred recurrently in TAL1 patients, 134 primary T-ALL samples were analysed by high-density Affymetrix SNP array-6 analysis; an ~700-bp region surrounding the Jurkat insertion site was also sequenced in a subset of 93 samples and six cell lines; in parallel, the literature was reviewed for cases with unexplained *TAL1* activation. While no macromolecular *TAL1* alteration was identified by SNP array (0/134), sequencing revealed seven new cases of similar microinsertions (1–9 bp), all precisely located at the Jurkat insertion breakpoint (Fig. 3a). Such insertions were not present in the germline from 2/2 available patients tested (Supplementary Fig. 4). No additional mutation/indel could be found in the surrounding ~700 bp region in tumour samples. Significantly, insertions were exclusively found in TAL1^+^ patients; moreover, among patients with informative SNPs in the *TAL1* 3′ UTR allowing distinction of mono- from biallelic expression^7^ (*n*=60), insertions were exclusively found in monoallelic cases (4/19, >20%), in agreement with a *cis*-mode of *TAL1* activation (Fig. 3b).

### Oncogenic RAG1/2-mediated episomal reintegration

**Figure 4:**
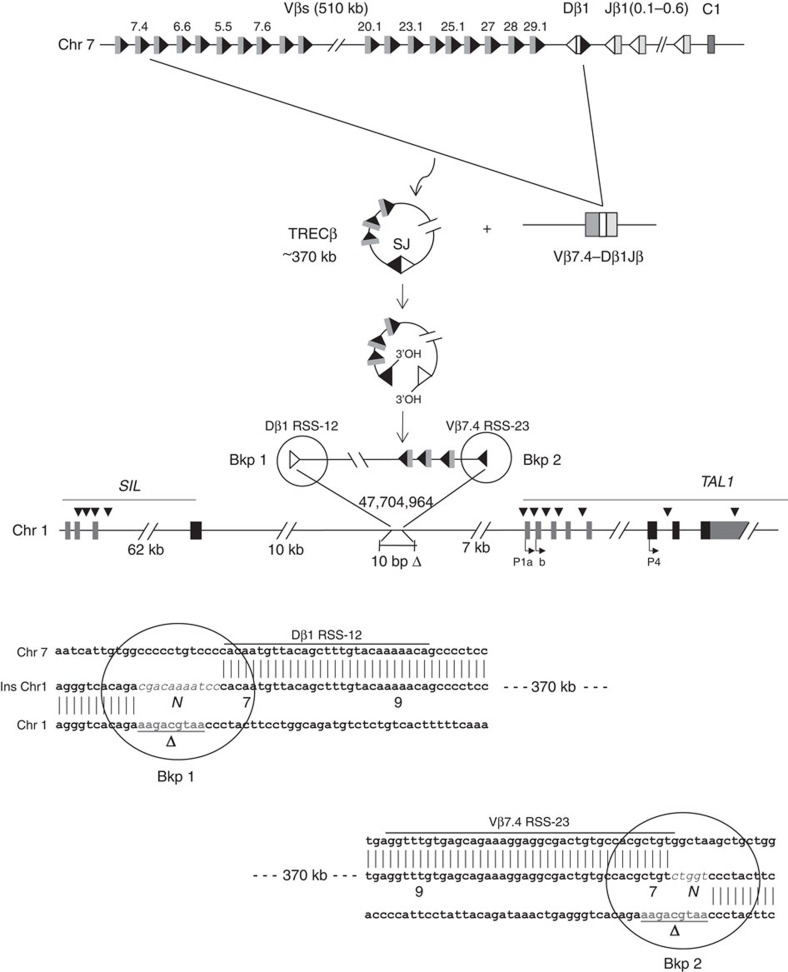
Schematic representation of the episomal reintegration in Patient OC. The TCRβ locus is displayed (top lane, not to scale). A functional Vβ7.4-to-Dβ1 rearrangement generating an excised TRECβ, and containing a (Vβ7.4/Dβ1) signal joint (SJ) is represented. The episome might have been open at the SJ by a nick–nick process^44^ generating 3′ hydroxyl ends before integration in chromosome 1. The episome is integrated in reverse orientation 10 kb downstream of the *STIL* gene, and 7 kb upstream of the *TAL1* gene (middle lane). A 10-bp deletion (Δ, underlined) occurred at the insertion site. Localization of cryptic RSSs used by illegitimate V(D)J-mediated SIL-TAL deletion, and by t(1;14) TCRδ/*TAL1* translocations are indicated by black arrow heads. *TAL1* promoters (P1a, P1b, P4) are indicated. The breakpoints sequences (Bkp1/2) are shown (bottom lane). *N*, N regions; Vβ7.4 and Dβ1 RSSs are indicated, with heptamers (7) and nonamers (9) depicted.

One additional candidate was recovered by data mining^16^. In this case (patient #OC), TAL1^high^ activation concurred with the insertion of a large piece of chromosome 7 disrupting the *TAL1* locus. Strikingly, breakpoint mapping by ligation-mediated PCR revealed that the insertion occurred at the very same insertion site, although this time with few nucleotide deletions on each side of the breakpoint (Fig. 3a). Detailed analysis of the junctions revealed the occurrence of RAG1/2-mediated reinsertion of an ~370-kb TCRβ episomal circle (TRECβ, excised during normal V(D)J recombination, Fig. 4 and Supplementary Fig. 5). This establishes the first example of oncogenic RAG1/2-mediated reintegration, demonstrating that TRECs may indeed contribute to oncogenesis^17^. Owing to the large TRECβ size, we considered the possibility that, unlike other microinsertions, a promoter located in the episome could have initiated a >7-kb-long fusion transcript encompassing *TAL1* (Supplementary Fig. 6). However, reverse transcription-PCR (RT–PCR) exon walking and 5′ RACE assays indicated that transcripts initiated from the *TAL1* p4 promoter, excluding this possibility.

### Epigenetic modulation and *TAL1* gene expression

**Figure 5:**
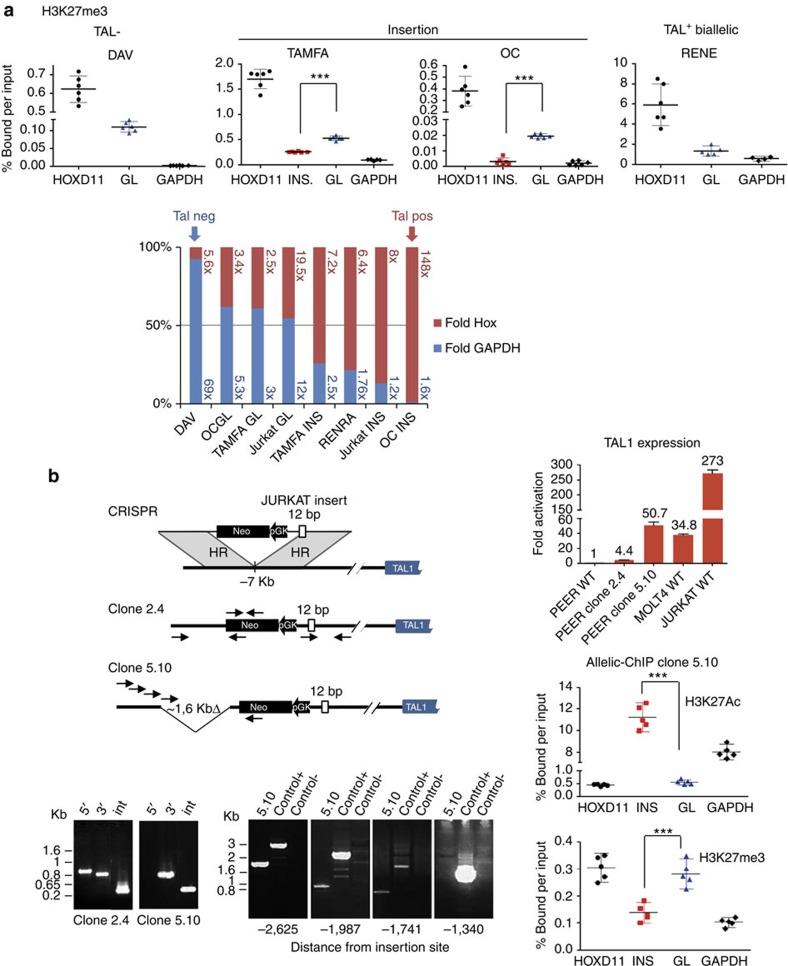
Insertional mutagenesis is associated with epigenetic modulation and *TAL1* gene expression. (**a**) Allelic-ChIP analysis of H3K27me3 marks at the insertion breakpoint in primary patients. See legend to Fig. 2a. Marks at the GL alleles in one TAL1^+^ biallelic (RENE) and in one TAL1^−^ (DAV) patients were performed as controls. ***P<0.001, unpaired *t*-test; Relative-fold plots: Inserted and/or GL allele ChIP values were calculated as fold increases relative to GAPDH (numbers in blue) or HoxD11 (numbers in red), and folds plotted as relative percentages (GAPDH relative folds: blue histograms; HOXD11 relative folds: red histograms). Blue histograms over 50% indicate higher differences with the expressed than the repressed control genes and correspond to (partially) repressed *TAL1* expression; conversely, red histograms over 50% indicate higher differences with the repressed than the expressed control genes and correspond to (partially) derepressed *TAL1* expression; histograms are ordered according to decreasing *TAL1* repression. (**b**) Epigenetic modulation and *TAL1* gene expression by DNA editing mimicking insertional mutagenesis. Left panel: schematic representation of the CRISPR design for homologous recombination at the *TAL1* locus, and configuration of two edited clones in the PEER cell line. The locations of PCR primers (plain arrows) for detecting successful targeted events and for genome walking are indicated. Bottom left panel: successful homologous recombination was confirmed by PCR of the expected genome-donor and donor-insert boundaries. Top right panel: RQ-PCR analysis of *TAL1* expression after editing. Transcripts were normalized to ABL and reported as relative values to non-edited PEER cells. Four PCR replicates were performed on 1 (clone 2.4, due to impaired growth) or 2 (clone 5.10) independent RNA extractions. Bottom right panel: allelic-ChIP assays of H3K27me3 and H3K27ac marks in edited clone 5.10. See legend to Fig. 2a. ***P<0.001, unpaired *t*-test.

Allelic-ChIP was then performed on patients #TAMFA and #OC. Similar to Jurkat, significant enrichment of the repressive H3K27me3 mark was consistently observed in germline compared with inserted alleles (Fig. 5a,b). The amplitude of the allelic distortion appeared higher in OC than in Jurkat and TAMFA, possibly due to the large difference in the insertion size. Of note, *TAL1* transcription levels were also higher in OC (Fig. 3b). Using CrispR DNA editing, we next mimicked site-specific insertion and disruption of the region 7 kb 5′ of *TAL1* in the TAL-negative PEER cell line (Fig. 5b). In clone #2.4 recapitulating the 12-bp Jurkat insertion at its 3′ end, an approximately fivefold increase of *TAL1* could be observed. While we cannot formally exclude the possibility that the selection cassette contributed to the fivefold change in clone #2.4, a 55-fold increase was observed in clone #5.10, in which an ~1.3 kb deletion 5′ of the insertion site mimicked locus disruption in patient #OC; furthermore, this was accompanied by an allelic switch from H3K27 methylation to acetylation. This provides direct evidence for a causal relationship between site mutagenesis, epigenetic modulation and *TAL1* gene expression.

### The mode of *TAL1* activation impacts on clinical outcome

**Figure 6:**
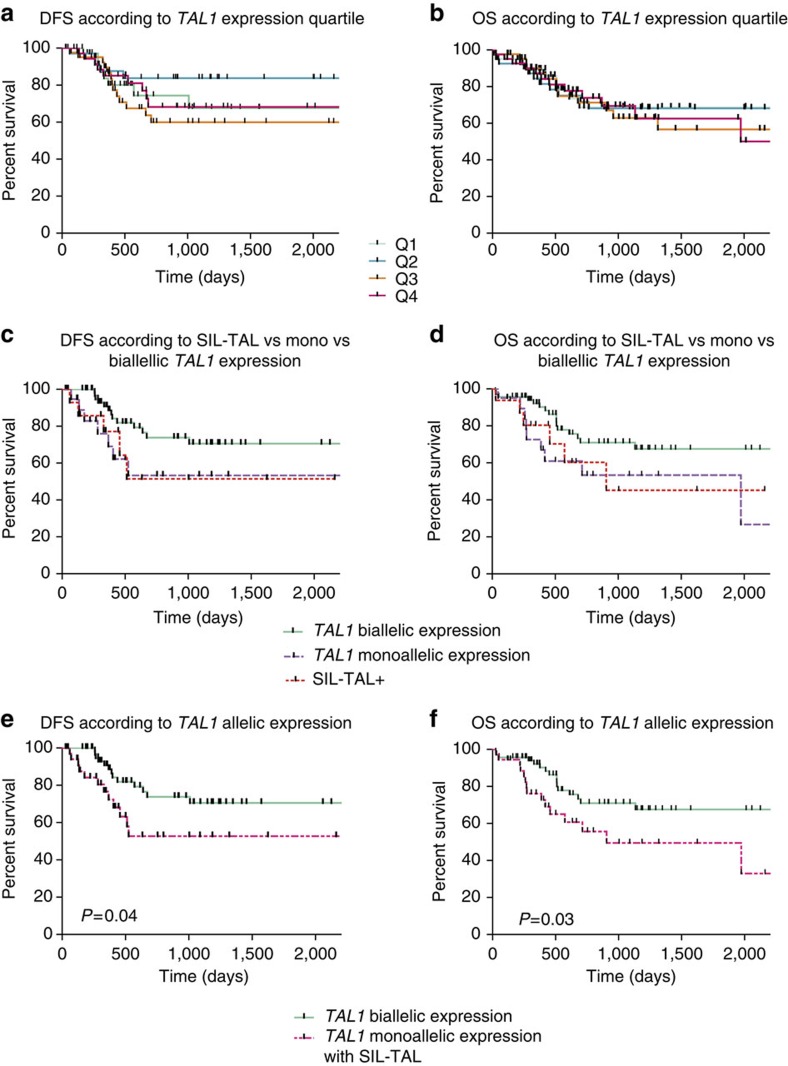
Survival analysis. Kaplan–Meier analysis showing DFS and OS of 165 protocolar patients treated in the GRAALL trial according to: (**a**,**b**) *TAL1* expression quartiles; (**c**–**f**) the mode of *TAL1* expression. *P* values are indicated, log-rank (Mantle–Cox) test.

Patients with identified insertions were globally of adverse prognosis. We sought to determine if clinical outcome correlated with quantitative or qualitative aspects of *TAL1* deregulation. A cohort of 165 adult T-ALL treated prospectively in the GRAALL (Group for Research in Adult Acute Lymphoblastic Leukaemia) trial was split into *TAL1* expression quartiles, and compared for disease-free (DFS) and overall survival (OS). The seven patients with identified insertions (three of whom were GRAALL treated), all belonged to the high-expression quartiles (Q3–4). However, no significant difference in survival was observed between the quartiles (Fig. 6a,b), suggesting that quantitative *TAL1* expression does not correlate with clinical outcome. We next tested whether *cis*-mediated *TAL1* alterations leading to monoallelic expression (including or not the SIL-TAL1^+^ cases) affected the clinical outcome compared with *trans*-mediated events, associated with biallelic *TAL1* expression. Clinical outcome was indeed found to be significantly improved in the biallelic group (DFS, *P*=0.04; OS, *P*=0.03; Fig. 6c–f). Although numbers are low, monoallelic cases retained an inferior OS trend in multivariate analysis (including age, leukocytosis; *P*=0.07, Cox analysis). Despite genetic heterogeneity, monoallelic cases also displayed higher blasts counts at diagnosis than biallelic cases and a significantly lower frequency of deregulation of recurrent oncogenes such as TLX1, CALM-AF10 and TLX3 (Supplementary Table 1). This supports the emerging notion that the mode of alteration may shape the oncogenic landscape in a more profound manner than (and potentially override the effect of) transcriptional levels, and that this may eventually impact on the tumour’s clinical behaviour^18^. Deciphering the mechanisms underlying the (epi)genetic deregulation of biallelic TAL1^+^ cases will be instrumental to resolving this issue^19^,^20^,^21^,^22^ (Supplementary Fig. 7 and Supplementary Table 2).

## Discussion

Establishing the detailed maps of the complex oncogenic networks involved in T-ALL has contributed to major genetic discoveries, and has been of prime importance for further therapeutic improvement. Over three decades of intense efforts in genomic research have allowed unravelling the extraordinary diversity of the mechanisms by which oncogenes are deregulated in this disease^1^. Yet, a large number of major oncogene deregulations still remain unexplained to date. Among the diversity of mechanisms involved, V(D)J recombination-mediated alterations (translocations, microdeletions) constitute the hallmarks of T-ALLs^23^,^24^,^25^,^26^. Interestingly, despite arrays of biochemical and functional evidence that the reintegration of excised episomal circles (TRECs) by the V(D)J recombinase (RAG1/2) might constitute a potent source of genomic instability, such events remained so far unreported in human cancer patients^8^,^9^,^27^,^28^,^29^,^30^. Here we report the first case of such an oncogenic RAG1/2-mediated episomal reintegration, demonstrating that TRECs can indeed contribute to human oncogenesis (Supplementary Fig. 1). Most intriguingly, this insertion occurred in a pediatric SCID-X1 patient who developed a leukaemia secondary to retroviral reinsertion (in front of LMO2, a known TAL1-cooperating oncogene) following gene therapy^16^. The screening of two other SCID-X1 patients’ leukaemic samples^31^,^32^, and of a large collection of T-ALLs did not reveal additional episomal insertions, and the reason for the extraordinary coincidence of two rare oncogenic integration events in this patient remains unanswered.

This and the other insertional mutagenesis T-ALL cases described here also revealed a novel oncogenic activation pathway, whereby a genetic alteration drives a site-specific and monoallelic epigenetic deregulation. We demonstrate that such insertions drove a switch from H3K27me3 to H3K27ac deposition, leading to the maintenance and/or re-expression of *TAL1* expression through T-cell differentiation. Interestingly, the difference in *TAL1* expression levels observed in mutants from the gene editing assay (Fig. 5) suggest that while small insertions might be sufficient to prevent the deposition of PcG repressive marks during T-cell lineage specification (thus permitting H3K27ac switch and maintenance of *TAL1* expression), further disruption of the region 5′ of the insertion site (by deletion or insertional uncoupling) might be necessary to impose desilencing once *TAL1* extinction is established in the T-cell lineage^6^ (Supplementary Fig. 1). These kinetics are coherent with thymocyte ontogeny in patient #OC, in which TRECβ rearrangement/reintegration (DN2-3) likely occurred after *TAL1* silencing (DN1-2). Altogether, our data are in line with current models of permanent gene extinction of transcription factors during T-lineage commitment^6^ (Supplementary Fig. 1) and further identify locus control regions involved in deposition and/or maintenance of *TAL1* silencing. Their genetic disruption constitute a recurrent epigenetic mechanism of *TAL1* deregulation in T-ALLs, contributing to a substantial fraction (>20%) of the TAL1^+^ monoallelic ‘unresolved cases’, and associated with adverse prognostic. That a *cis-*deregulation regrouping as diverse mechanisms as SIL-TAL deletions, translocations or insertional desilencing impact more on prognosis than *TAL1* expression levels underlines the fundamental oncogenic difference between a deregulation targeting a single locus, and the wider effect of *trans*-acting factors. Transcription factors indeed often bind to a large number of target genes (hundreds to thousands) and their deregulation (whether gain or loss) will likely affect a complex set of cellular functions, some of which might antagonize tumour progression, or resistance to treatment. Recently, reports identifying loss-of-function mutations in polycomb-related components^19^,^20^,^21^,^33^ have provided the framework by which global epigenetic modification might trigger the indirect (and biallelic) activation of numerous target genes, likely including a complex and conflicting set of oncogenes and tumour suppressors. In humans, PcG are recruited to and repress specific regions in the genome through as yet undefined set(s) of DNA-binding transcription factors and long non-coding RNAs^34^. The insertional mutagenesis described here identifies a site- and allele-specific switch from H3K27me3 recruitment/maintenance to H3K27ac, providing new avenues to decipher the mechanisms and DNA-binding intermediates involved^3^. A complex interplay between transcription factors and PcG would be in line with recent findings that Notch1 activation antagonizes PRC2 silencing of Notch1 target genes in T-ALL oncogenesis^19^. In intricate T-ALL networks where both *NOTCH* and *TAL1* deregulation can coexist, we find that while NOTCH patients are associated with a favourable prognosis, monoallelic, but not biallelic *TAL1*, expression tends to convert the clinical outcome towards more adverse prognosis in the Notch subgroup, and to further aggravate the bad prognosis of Notch1^WT^ patients. This suggests that distinct modes of deregulation of the same epigenetic complex might coexist in a tumour cell, leading to complex and potentially conflicting clinical outcomes which ought to be clarified when considering epigenetic inhibitors for new lines of treatment^15^,^33^,^35^.

## Methods

### ChIP

ChIP was performed as previously described^10^. In brief, the cells were chemically crosslinked by the addition of one-tenth volume of fresh 11% formaldehyde solution for 10 min at room temperature. Following the quenching of the reaction with glycine (250 mM final concentration, 5 min, room temperature), cells were rinsed twice with 1 × PBS and flash frozen in liquid nitrogen and stored at −80 °C before use. Cells were resuspended, lysed and sonicated to solubilize and shear crosslinked DNA. Sonication was conducted using a Bioruptor (Next Gen, Diagenode) for 15 min (30 s on, 30 s off), resulting in sheared DNA between 100 and 400 bp with the bulk at ~250 bp. The resulting whole-cell extract was incubated overnight at 4 °C with 100 μl of Dynal Protein G magnetic beads that had been preincubated with the appropriate antibody. The anti-H3K27me3 antibodies used were: # 07-449, Millipore (2 μg), and # ab6002, Abcam (1 μg); the anti-H3K27ac antibodies used were: #ab4729, Abcam (2 μg) and # 39133, active motif (5 μg). Beads were washed eight times with RIPA buffer and one time with TE containing 50 mM NaCl. Bound complexes were eluted from the beads by heating at 65 °C with occasional vortexing, and crosslinking was reversed by overnight incubation at 65 °C. Whole-cell extract DNA (reserved from the sonication step) was also treated for crosslink reversal. Immunoprecipitated DNA and whole-cell extract DNA were then purified by treatment with RNaseA, proteinase K and multiple phenol:chloroform: isoamyl alcohol extractions.

### ChIP-seq

Before sequencing, ChIP DNA was quantified using the picogreen method (Invitrogen, USA) and quality controlled on a 2100 Bioanalyzer (Agilent). At least 1 ng of ChIP or input DNA was used for library preparation according to the Illumina ChIP-seq protocol. After end repair and adapter ligation, fragments were size selected on a gel before preamplification and clustering. The resulting fragments were again verified on a 2100 Bioanalyzer before clustering and 25 or 36 cycle sequencing on a Genome Analyzer II (Illumina, USA) according to the manufacturer’s instructions. Raw data were bowtie aligned and the tags elongated and further processed to wiggle files as described^36^. ChIP-seq experiments were performed on two independent biological replicates (samples for primary cells or culture for cell lines). Data presented in Fig. 1 are available under accession number GSE29362 for TBP, Pol II, H3K4me1, H3K4me3 and H3K36me3 or GSE38577for H3K27me3(1A), and for H3K27me3 in human cells (1B) GSE12646 for HSC and erythroblast, GSE12889 for CD4+ and GSE59257 for the data newly generated (Jurkat and DA) in this article. Technical replicates were merged before alignment with Bowtie and the resulting BAM files were used as treatment in MACS2. Input data sets were used as control when available (Jurkat, DA). Enriched regions for genomic tracks of Fig. 1a,b were extracted as BED files using MACS2 peak detection algorithm with the following parameters : genome sizes=2.70e+09 for human and 1.87e+09 for mouse, bandwidth=300, model fold=(5, 50), *q* value cutoff=5.00e−02, larger scaled towards smaller, *λ* range=1,000–10,000 bps, Broad on.

### Assessment of wt versus inserted ChIP-seq signal

To identify a difference in H3K27ac enrichment in ChIP-seq between alleles with and without insertion (12 bp) in Fig. 2b, we aligned all 25 bp reads of each experiment to this specific region of insertion using the R package ‘Biostrings’. This alignment was performed against a region of 52 bp around the insertion site allowing for up to two mismatches, and the number of hits on forward and reverse strands was cumulated. For the mutant allele, the Jurkat insert sequence (5′-CCGTTTCCTAAG-3′) was inserted in the reference sequence before alignment, extending the initial alignment region to a size of 64 bp.

### Allelic-ChIP

Input and IP genomic DNAs were analysed by RT–PCR using power SybrGreen on a 7,500 Fast Real-time PCR system (Applied Biosystems). IgG control ‘cycle over the threshold’ Ct values were subtracted to Input or IP Ct values and converted into bound value by 2^−(IP Ct or input Ct- IgG IP Ct)^. Allelic-ChIP was carried out using allele-specific primers (by substituting one of the germline primers with a primer located in or overlapping the insertion). EZH2 knockdown was achieved using a doxycycline-induced short hairpin RNA (sh-RNA)-targeting EZH2 (pTRIPZ-EZH2, openbiosystem # V21HS-63066). A non-silencing sh-RNA (pTRIPZ-NS) was used as control. Jurkat cells were electroporated and cells containing the pTRIPZ were selected on puromycine. Knockdown of EZH2 was obtained by the addition of doxycycline (2 μg ml^−1^) to the cells during 10 days. Western blot was performed using the anti-EZH2 BD Biosciences # 612666.

### Sequencing and SNP array

For Jurkat mapping, a region of 10 kb 5′ of *TAL1* exon 1 was mapped on both alleles by LRPCR/cloning and standard Sanger sequencing as previously described^37^ (see Supplementary Fig. 2 for details on mapping strategy). Identification of allelic variants (SNPs versus somatic indels/mutations) was performed with vector NTI using alignment against reference *TAL1* sequence and variants (http://www.ncbi.nlm.nih.gov/SNP; http://projects.tcag.ca/variation, Supplementary Fig. 8). For the sequencing screen on T-ALL patients and cell lines, a region of ~700 bp surrounding the Jurkat insertion site was directly PCR/sequenced on both strands in a subset of 93 samples and six cell lines. Heterogeneous sequences (ambiguous reading due to allelic differences) were systematically cloned, sequenced and analysed as above. For SNP array, hybridization on Affymetrix Cytogenetics SNP Array-6 was performed according to the manufacturer’s recommendations. Data analysis was performed with Chromosome Analysis Suite software using the following settings: the CGH log2 copy number ratio for heterozygous deletion was defined as 0.5 to 1.5, whereas log2 copy number ratios <1.5 were defined as homozygous deletions. Gene copy number (GCN) aberrations were compared with the Database of Genomic variants (http://projects.tcag.ca/variation) to study only non-variant GCV aberrations.

### Patients

Diagnostic samples from a consecutive series T-ALLs from 165 adults (older than 16 years) included in GRAALL-03/05 trial (registration #NCT00327678 and #NCT00222027) were analysed for *TAL1* expression. Sample collection and analyses were approved by the local ethical committee. Informed consent was obtained from the patients or relatives in accordance with the Declaration of Helsinki, with the institutional review board approval of all involved hospitals. Diagnosis of T-ALL was based on the World Health Organization 2008 criteria, defined by expression of cytoplasmic and/or surface CD3, and negativity of CD19 and MPO, as reported^38^. To be included, samples had to contain at least 80% of lymphoblasts. Immuno-geno/phenotyping and oncogene quantification were performed as previously described^38^,^39^.

### Cell lines

Cell lines used in this study were purchased from the ATCC collection and were mycoplasma free.

### RQ-PCR

RNA was reverse transcripted using MMLV (Invitrogen). We used a TaqMan assay to quantify *TAL1* transcript with the following primers: TAL1 F: 5′-ACA-ATC-GAG-TGA-AGA-GGA-GAC-CTT-C-3′, TAL1 Probe: fam-5′*-*CTA-TGA-GAT-GGA-GAT-GGA-GAT-TAC-TGA-TG-3′-tamra, TAL1 R: 5′-ACG-CCG-CAC-AAC-TTT-GGT-G-3′, 40 cycles were run on ABI 7500HT (Applied Biosystem) as described^40^. *TAL1* transcript quantification was performed after normalization with the housekeeping gene *ABL* using the ΔCt method and results calculated according to the following formula 2^Δ(CtABL–CtTAL1)^.

### *TAL1* allelic expression analysis

Allelic expression was performed as previously described^7^. In brief, polymorphic markers in the 3′ UTR of the *TAL1* gene were identified by PCR amplification and direct sequencing of 100 ng of genomic DNA. Allelic expression analysis was performed by PCR amplification and by direct sequencing of RT–PCR products from heterozygous patient samples. Three different PCRs were made to cover nine most frequent SNP among the 11 SNP previously described^7^.

### Statistical analysis

*ChIP*. The power of *t*-test was estimated a priori using pwrR-package, and the expected variations between conditions evaluated. Differences in ChIP data between inserted and GL *TAL1* alleles were analysed by unpaired *t*-test. Samples collections constituted of five or six technical replicates were first checked for normal distribution using Kolmogorov–Smirnov test and the equality of variances was tested using F test. Results of *t*-test are shown as two-tailed *P* values. The statistical power of executed *t*-tests was at least 80%. Errors bars on histograms represent s.e.m.

*OS/DFS*. Patients’ characteristics were compared using the Fisher’s exact test. Median comparisons were performed using the Mann–Whitney *U-*test. OS and DFS were calculated from the date of prephase initiation. Events accounting for DFS were induction failure and first relapse from any cause in first CR. OS and DFS were estimated by the Kaplan–Meier method and then compared by the log-rank test. All calculations were performed using the SPSS software, version 15.0 and the GraphPad Prism, La Jolla, CA, USA.

### Ligation-mediated PCR

Genomic DNA was extracted using QIAamp deoxyribonucleic acid Blood mini kit according to the manufacturer’s instructions (Qiagen). DNA (500 ng) was used for LM-PCR; DNA was digested by DraI, EcoRV, PvuII, SmaI, SspI or StuI restriction endonucleases. Purified digested DNAs were ligated with an adapter (composed of two complementary primers GWA^+^ and GWAB^−^). A primary PCR amplification was performed using an adapter-specific primer (AP1) and primer specific for the different TCRβ gene segments (ext). A secondary PCR was performed using nested AP2 and TCRβ primers (int), and analysed on 1% agarose gel. Non-germline PCR products were purified and sequenced. The functional and non-functional V(D)J rearrangements from patient OC were obtained using Jβ2.7-extB/intB and AP1/2 primers. The breakpoints corresponding to the episomal insertion were then obtained using Vβ7.4-extB/intB, Dβ1-extA/intA and AP1/2 primers, and validated by direct PCR using TAL1.OC.1B and Vβ7.4-intB primers.

### RT–PCR exon walking and 5′ RACE

Total RNA was extracted using a column-based system RNAeasy mini kit (Qiagen) according to the manufacturer’s instructions. Reverse transcription was performed with SuperScript III Reverse transcriptase (Invitrogen) and random primers (applied Biosytem). cDNAs were analysed by real-time quantitative PCR (RT–PCR) using power SybrGreen on an ABIPRISM 7500 (Applied Biosystems). All PCRs were performed in duplicate. 5′-RACE was performed using 2 μg of total RNA and the 5′/3′ RACE kit, 2nd generation (Roche). Modifications from the instruction manufacturer were the generation a poly(G) tailing of first strand cDNA and the use of an oligo d(C) anchor primer. PCR was performed using the Pfu Ultra II fusion HS DNA polymerase (Agilent technologies).

### Genome editing in T-ALL cell line by type II CRISPR system

PEER T-ALL cells line were cultured in RPMI medium (Life Technologies) containing 20% fetal calf serum, 1% L-glutamine, 1% sodium pyruvate and 100 U ml^−1^ penicillin/streptomycin (Life Technologies) at 37 °C in the presence of 5% CO2. The day of transfection, 1 million cells were nucleofected according to the manufacturer’s instruction (Lonza), with 500 ng DNA donor sequence containing Neomycin-resistant gene and 2 μg of the Cas9/gRNA expression vector (Addgene #42230). The chimeric guide RNA targeted *TAL1* insertion site, and was cloned according to Cong *et al.*^41^. One day after nucleofection, cells were plated in 96-wells plate at 10^4^ cells per well and incubated in presence of 1,200 μg ml^−1^ geneticin G418 (Life Technologies) for 2 weeks. After selection and growing, a PCR was conducted to amplify the targeted region with genomic DNA derived from the surviving clones, and amplicons were separated on a 1% agarose gel then extracted with GEL/PCR clean up wizard (Promega) and sequenced (MWG-Biotech). CRISPR guide RNA: 5′-GAAAGACGTAACCCTACTTCC-3′.

Primers list is available on request.

## Author contributions

ChIP-seq experiments were performed and analysed by L.C.P., R.F., M.A.M., M.G., I.G.G. and J.-C.A.; allelic-ChIP experiments were performed and analysed by J.-M.N., L.C.P., M.L., M.K., C.P., D.P.-B. and E.D.; T-ALL sample characterizations were performed and analysed by A.T., J.-M.N., L.C.P., S.L.N., S.J., L.S., E.A.M. and V.A.; TALEN/CRISPR assays were performed and analysed by J.-M.N., M.L. and D.P.-B. with the advice of C.G. and B.M.; episomal reintegration characterization was performed by J.-M.N., E.A.M., S.H.-B.-A., S.J.H., H.B.G. and A.J.T.; survival analysis was carried out by A.T., H.D., E.A.M., N.I. and V.A.; V.A. and B.N. conceived and directed the project, and B.N. wrote the paper.

## Additional information

**How to cite this article**: Navarro, J.-M. *et al.* Site- and allele-specific polycomb dysregulation in T-cell leukaemia. *Nat. Commun.* 6:6094 doi: 10.1038/ncomms7094 (2015).

## Supplementary Material

Supplementary Information Supplementary Figures 1-8, Supplementary Tables 1-2 and Supplementary References
