# Supplementary material for: Site- and allele-specific polycomb dysregulation in T-cell leukaemia
Source: Nat Commun. 2015 Jan 23;6:6094. doi: 10.1038/ncomms7094 (PMC4317503; doi:10.1038/ncomms7094)
Supplement: Supplementary Information — Supplementary Figures 1-8, Supplementary Tables 1-2 and Supplementary References [file ncomms7094-s1.pdf]

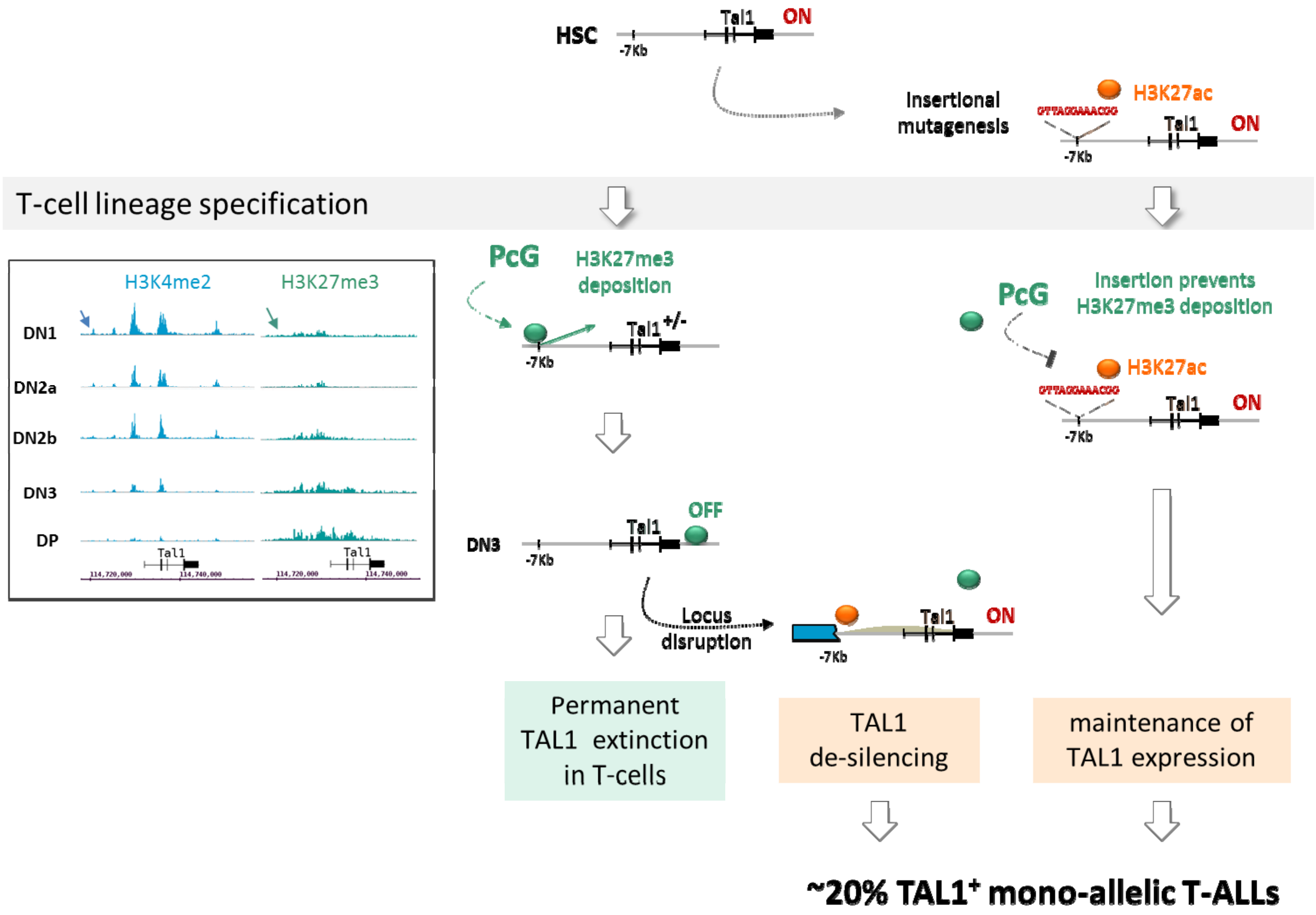
HSC
Tal1
ON
-7Kb
Insertional mutagenesis
H3K27ac
GTTAGGAAACGG
T-cell lineage specification
H3K4me2
H3K27me3
DN1
DN2a
DN2b
DN3
DP
PcG
H3K27me3 deposition
Tal1 +/-
Insertion prevents H3K27me3 deposition
OFF
Locus disruption
Permanent TAL1 extinction in T-cells
TAL1 de-silencing
maintenance of TAL1 expression
~20% TAL1+ mono-allelic T-ALLs

Supplementary Figure 1: Model of TAL1 extinction in T-cell specification, and dysregulation through maintenance/de-silencing in T-ALL. TAL1 is a regulatory gene that promotes access to alternative fates in hematopoiesis. Expressed in HSC (top panel), TAL1 expression is maintained in the erythroid lineage, but irreversibly silenced in the T-cell lineage. Silencing occurs through *de novo* H3K27me3 marking at a previous active TSS/enhancer[1]. Extending on data from Rothenberg and coll., our further analysis shows a first focal deposition of H3K27me3 at the 5' side of the locus (from the insertion site to p2) around DN1/DN2a, followed by propagation on the gene body at DN2, to reach complete extent of the mark at DN3/DP (Left panel, ChIP-seq). In this model (right panel), insertional mutagenesis before T-lineage specification would prevent the transcriptional repression and/or PcG focal deposition, leading to the maintenance of TAL1 expression similarly to what occurs in the erythroid lineage. Following PcG-mediated repression, further locus disruption (insertional/deletional mutagenesis) would be required to allow de-silencing through a switch from histone methylation to acetylation. Subsequent TAL1 expression in developing T-cells would contribute to T-ALL development.

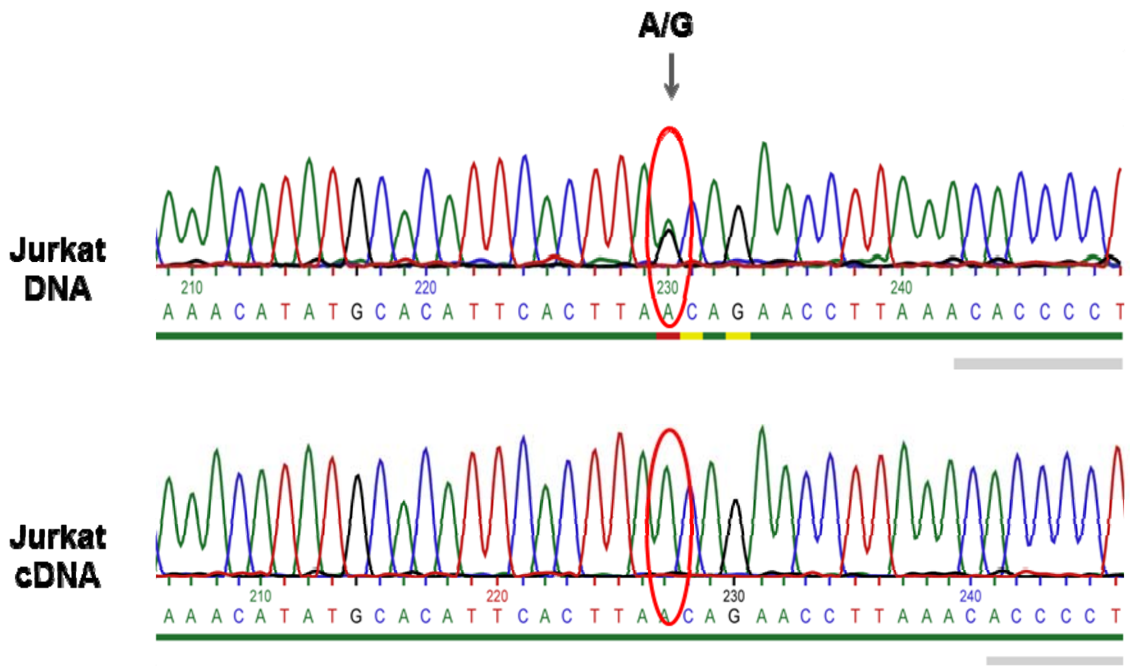

cDNA	« A » Allele	« G » Allele
cloning/seq	68/70 (97%)	2/70 (3%)

Supplementary Figure 2 Monoallelic TAL1 expression in Jurkat cells. Top panel: DNA sequence of the TAL1 3‘ UTR region in Jurkat, in which an informative A>G SNP allows to differentiate the “A” allele from the “G” allele. Bottom panel: cDNA sequence of the same region reveals monoallelic expression of the “A” allele; amplicons were cloned and sequenced to further quantify the allelic ratio. The “A” allele is preferentially expressed (97%) over the “G” allele.

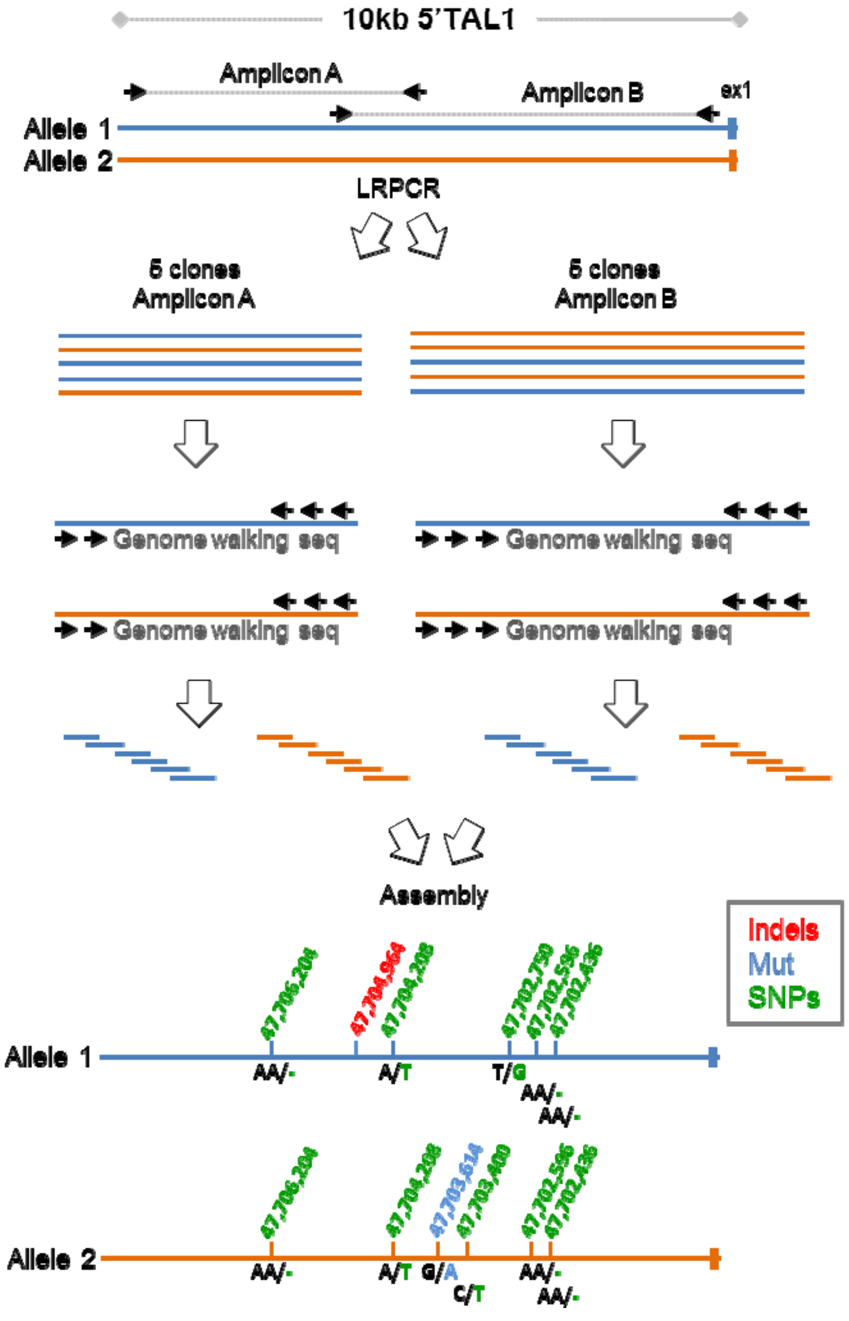

Supplementary Figure 3: 5'TAL1 sequence mapping strategy in Jurkat. A region of 10kb 5' of TAL1 exon 1 was mapped on both alleles by PCR/cloning/sequencing. Two overlapping genomic DNA fragments (A = 4.33 kb and B= 7.6 kb) were amplified from Jurkat cells. Five of each amplicons were cloned, sequenced using "genome-walking" primers, and allelic variants mapped on each allele. The presence of allelic variants in the region overlapping the two amplicons allowed assembly of the whole 10kb fragment on each allele. The position of Indels (red), somatic mutations (Mut, blue), and common SNPs (green) in each allele are indicated (HMG19 coordinates). All variants from the reference sequence were subsequently validated by short-range PCR/direct sequencing on Jurkat DNA.

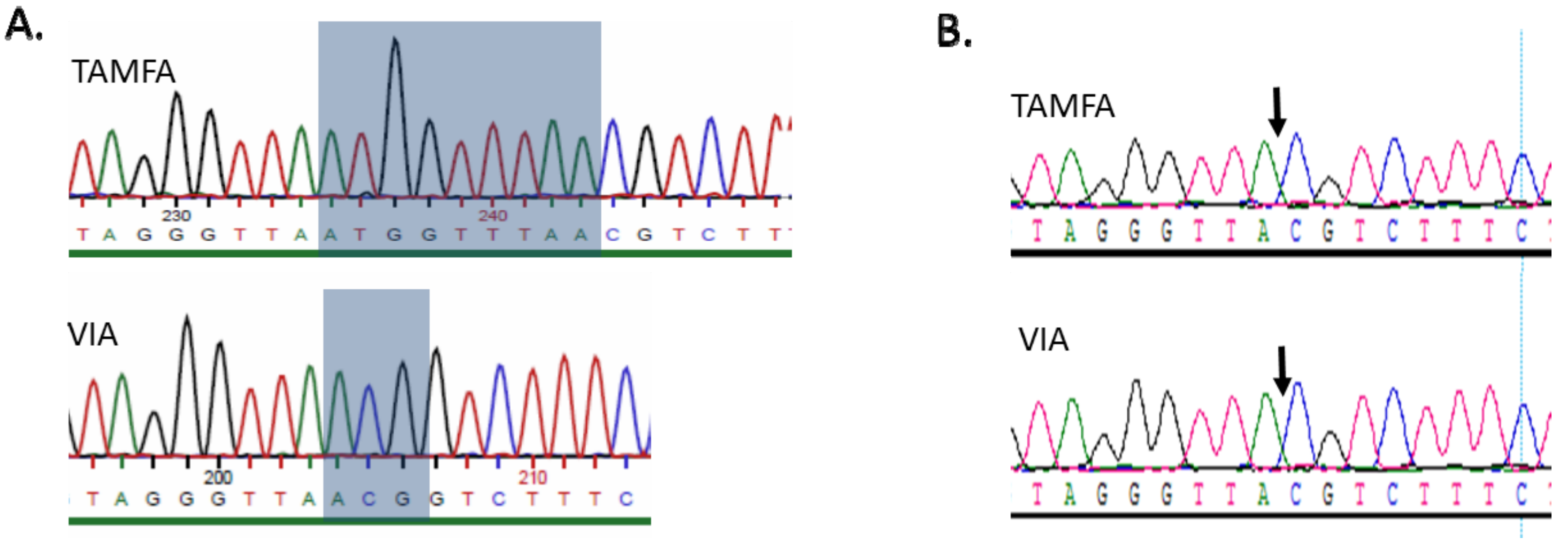

Supplementary Figure 4: Insertions upstream of *TAL1* are somatic events. Sequences of *TAL1* inserted regions from 2 T-ALL patients: A. in tumor sample; B. in bone marrow at remission. At remission, the proportion of blasts in the bone marrow was below 0,01%. Inserted sequences are highlighted by a blue box. Vertical arrows indicate the site of insertion.

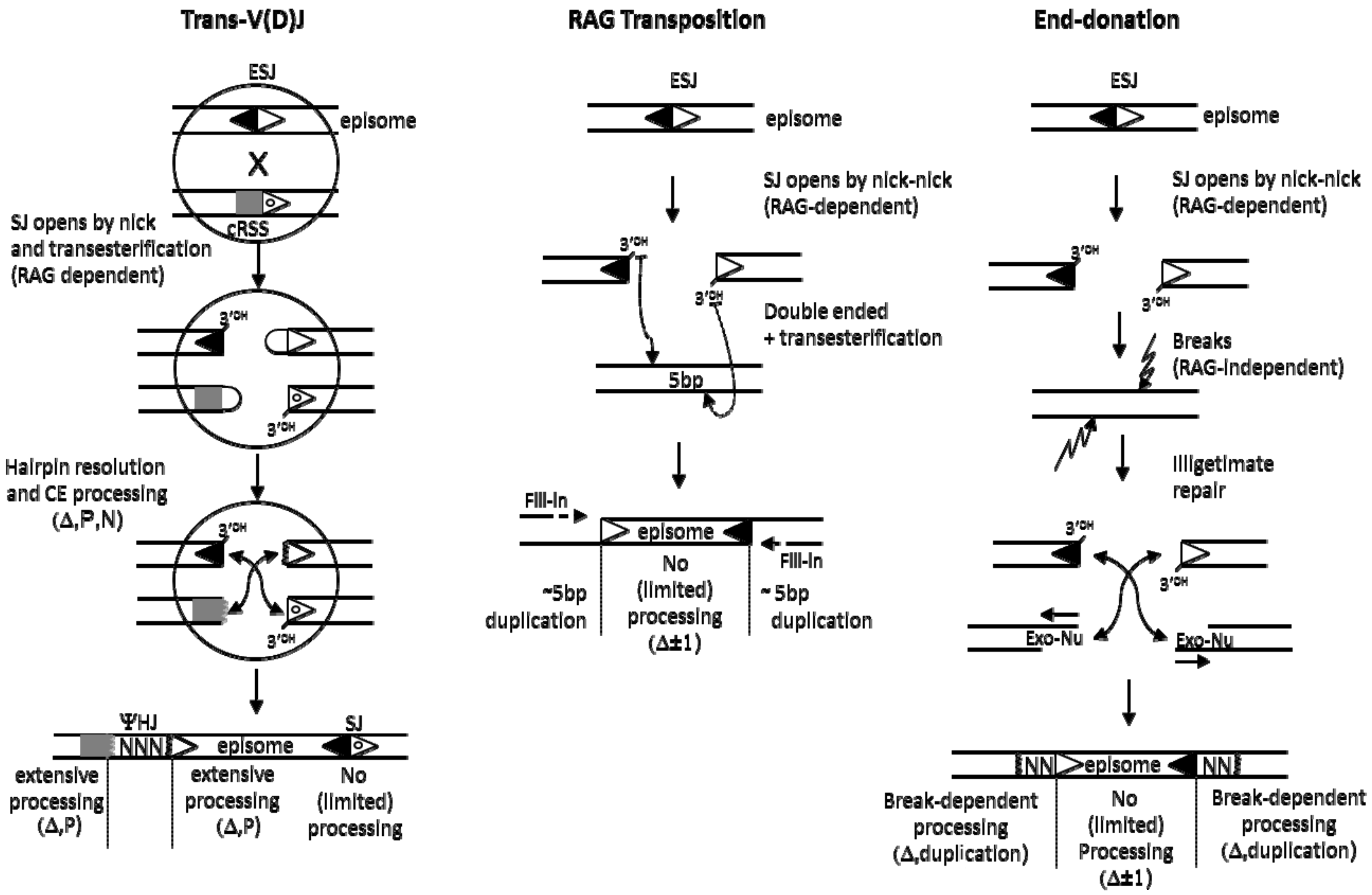
Trans-V(D)J
RAG Transposition
End-donation
ESJ
episome
X
cRSS
SJ opens by nick
and transesterification
(RAG dependent)
3'OH
Hairpin resolution
and CE processing
(Δ,P,N)
ΨHJ
NNN
SJ
extensive
processing
(Δ,P)
No
(limited)
processing
SJ opens by nick-nick
(RAG-dependent)
Double ended
+ transesterification
5bp
Fill-in
~5bp
duplication
(Δ±1)
Breaks
(RAG-independent)
Illigetimate
repair
Exo-Nu
Break-dependent
processing
(Δ,duplication)
Processing

Supplementary Figure 5: RAG1/2-mediated episomal reinsertion mechanism in patient OC. A. Schematic representation of 3 potential RAG-mediated insertion mechanisms involving the episomal SJ (ESJ), with expected genomic imprints. The precise breaks at the RSS borders in patient OC indicate that a RAG-mediated cleavage of the ESJ was likely involved in the opening of the episome. At least three mechanisms can account for subsequent episomal reinsertion[2]**: 1. *Trans*-V(D)J recombination**[3]: this mechanism involves synapsis of the ESJ with a cryptic RSS at the insertion site. Ongoing SJ recombination (including insertion through *trans*-V(D)J recombination) generates two characteristic breakpoint signatures[3,4]: one novel SJ and one particular hybrid RSS/coding-segment junction called "ΨHJ", in which both the coding end and the RSS partners undergo RAG-mediated cleavage (nucleophile trans-esterification, hairpin formation), processing (Artemis-dependent hairpin cleavage, N nucleotide insertion, nucleotide deletion, P nucleotide addition) and sealing. As depicted in Fig.4, analysis of OC breakpoints revealed neither the presence of hallmark SJ and ΨHJ, nor the presence of cryptic RSS sequence (defined by the canonical ↓CACNNNN cryptic heptamer) immediately 3' of the insertion site (RIC scores[5] failed, not shown). As we and others have previously reported functional non-canonical cryptic sites devoid of the CAC trimer[6], we further functionally validated the absence of RAG-mediated recombinogenic activity of the insertion site and surrounding sequences in *ex vivo* extra-chromosomal recombination substrates (not shown). That the insertion occurred outside a cryptic site is surprising considering the many functional cryptic RSSs surrounding the insertion region (Fig.4), some of which largely documented to be involved in RAG-mediated STIL-TAL1 deletion or t(1;14) translocation. **2. RAG-mediated** transposition[7,8]: double-ended RAG-mediated transposition is an alternative mechanism of episomal insertion which does not involve RSS at the insertion site. This mechanism generates specific imprints at the breakpoints, namely a short (~5bp) duplication of the insertion site, due to the asymmetric nucleophile attack of episomal SEs leaving staggered-type opening of the genomic target, subsequently filled-in during

ligation/repair. No such transposition marks were apparent at the breakpoints in OC patient (Fig.4); **3. End-donation**[9]: end-donation is another mechanism of episomal insertion which does not involve RSS at the insertion site. Intermediate coding or signal[10](RSS) ends are erroneously repaired with chromosomal broken-ends, and breakpoint features include N nucleotide addition in the junction; the presence of short deletions, or duplications at the broken end side depends on whether the initial break generated 3' overhang, blunt or 3' recessed ends. End-donation is by far the most frequent mechanism of V(D)J-mediated translocations in human B- and T-cell neoplasia (also called "type 2" translocation), and a frequent mechanism of episomal reinsertion in lymphoid cells from mice models (estimated to occur once every ~50,000 V(D)J recombinations)[11,12]. Breakpoint features in OC patient were compatible with end-donation. Intriguingly, this case corresponds to a SCID-X1 patient who developed leukemia secondary to another insertion (a retroviral-induced insertion in front of LMO2, a well-known TAL1-cooperating oncogene, following gene therapy).

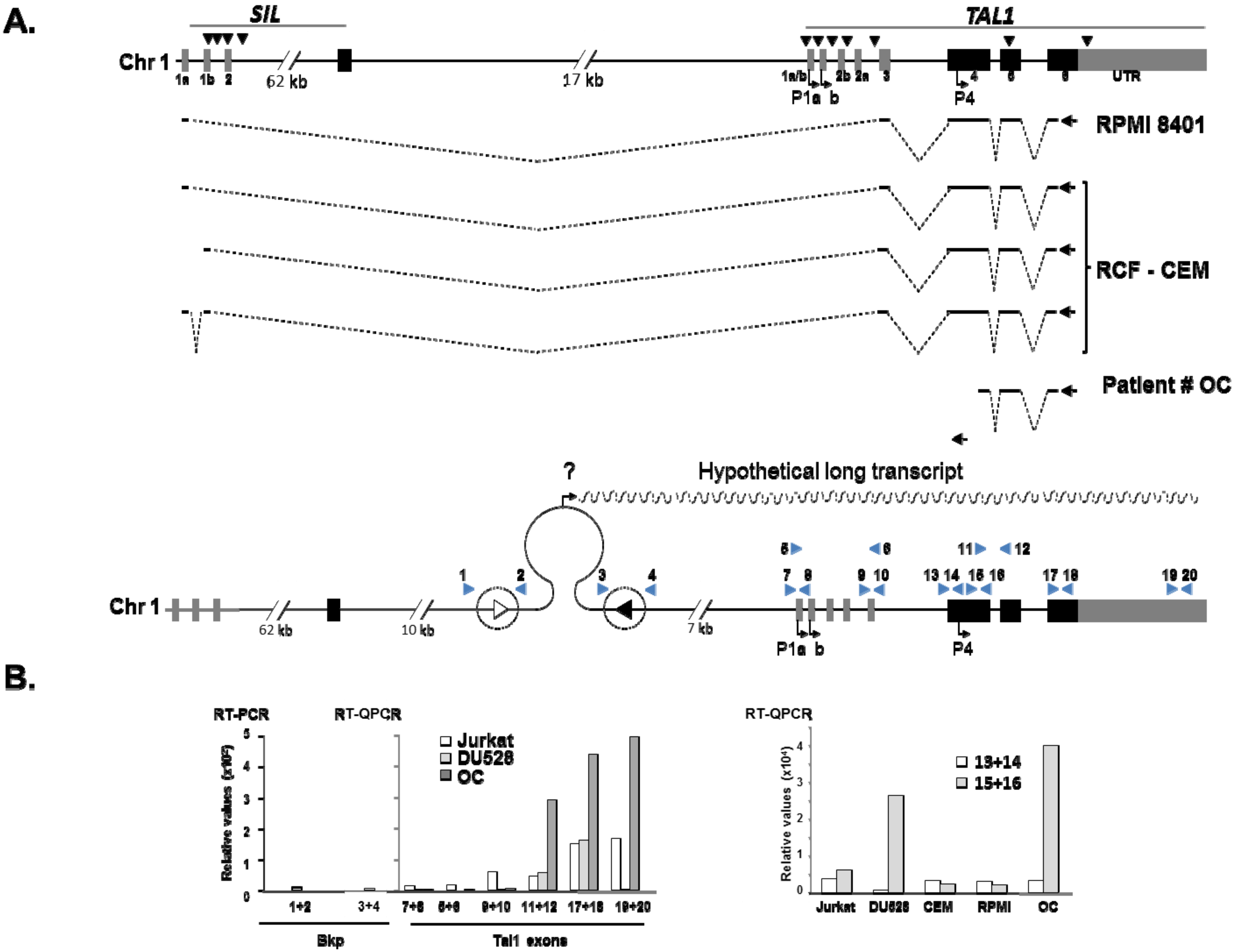
A.
SIL
TAL1
Chr 1
1a 1b 2
62 kb
17 kb
1a/b
2b 2a 3
4 5 6 UTR
P1a b
P4
RPMI 8401
RCF - CEM
Patient # OC
?
Hypothetical long transcript
1 2 3 4
5 6 7 8 9 10
11 12 13 14 15 16 17 18 19 20
10 kb
7 kb
B.
RT-PCR
RT-QPCR
Relative values
Jurkat
DU528
OC
1+2
3+4
7+8
5+6
9+10
11+12
17+18
19+20
Bkp
Tal1 exons
13+14
15+16
CEM
RPMI

Supplementary Figure 6. Identification of TAL1 transcription start site in Patient OC. **A.** A putative mechanism in which transcription initiates from the episome, and generates a >7kb-long fusion transcript encompassing TAL1 (illustrated in the bottom lane) was tested. A Rapid Amplification of cDNA Ends (5' RACE) assay was performed from TAL1 exon 4 and exon 6. STIL-TAL1 cell lines (RCF-CEM and RPMI 8401) in which transcription initiates from STIL promoters, were used as controls, and gave rise to complete SIL-TAL fusion transcripts (top lanes). In OC, a single transcript corresponding to the oncogenic p4 TAL1 variant was obtained from exon 6 (starting from p4 and comprising part of exon 4, and full exons 5 and 6), and accordingly no RACE product could be obtained from exon 4 (middle lanes). RACE primers are indicated by black arrows. **B.** A RT-PCR exon walking assay was also performed in and between various TAL1 exons, across the episomal breakpoint, and in the episome to detect potential splice variants. Expression is normalized to ABL. In line with the RACE data, no amplification of TAL1 exons or breakpoints was observed upstream of p4 (walking primers are pictured as blue arrow-heads). We conclude that TAL1 overexpression in OC was not initiated from a transcriptional start site located in the inserted episome.

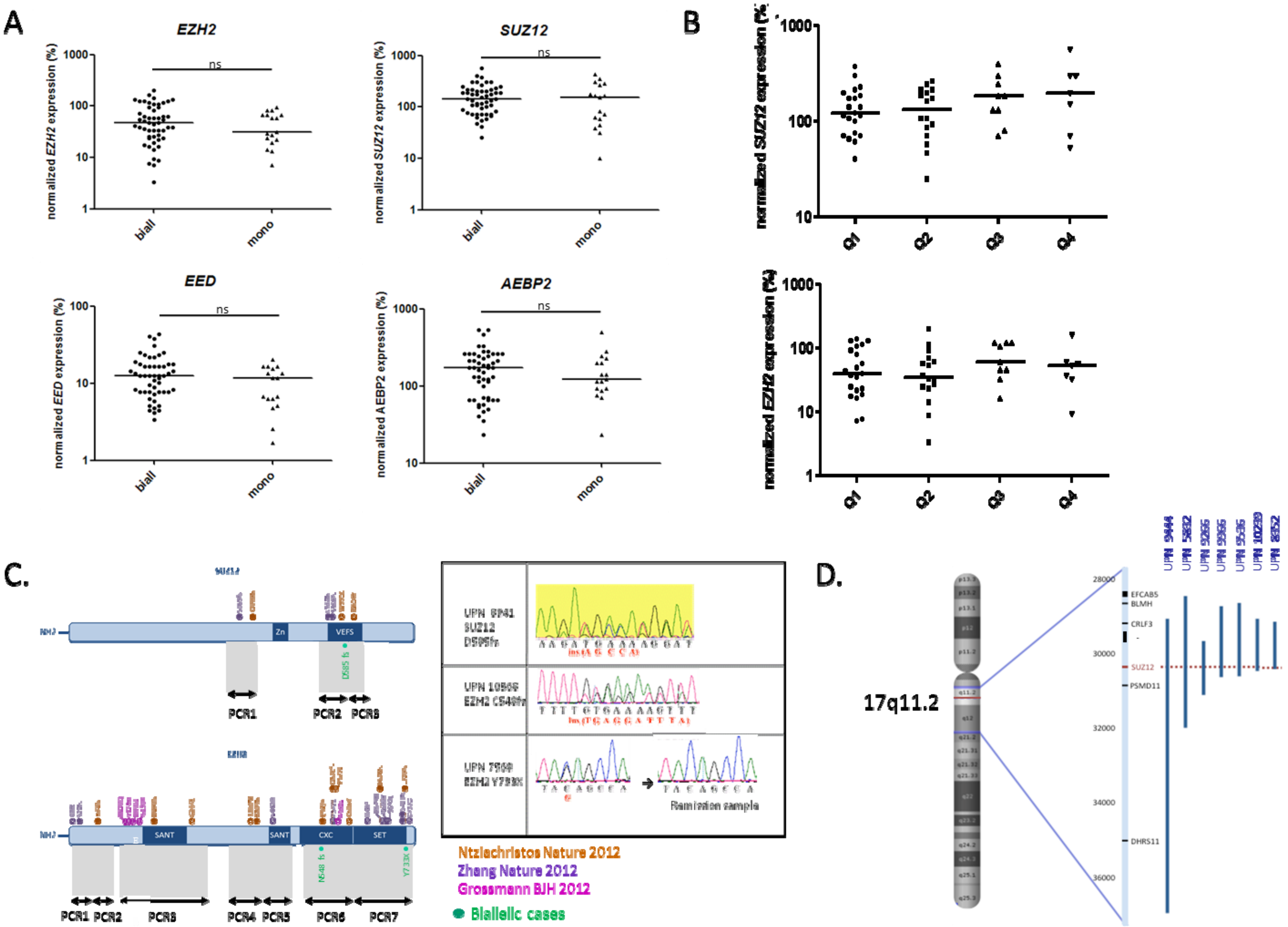
A
EZH2
SUZ12
EED
AEBP2
ns
biall
mono
B
Q1
Q2
Q3
Q4
C.
PCR1
PCR2
PCR3
PCR4
PCR5
PCR6
PCR7
SANT
CXC
SET
VEFS
Zn
Remission sample
Ntziachristos Nature 2012
Zhang Nature 2012
Grossmann BJH 2012
Biallelic cases
D.
17q11.2
SUZ12
PSMD11
DHRS11
CRLF3
BLMH
EFCAB5

Supplementary Figure 7: Dysregulation of PcG complex in bi-allelicTAL1 cases. Considering the involvement of PcG in insertional mutagenesis leading to mono-allelic TAL1 expression, we investigated whether PRC2 mutations might similarly lead to trans-activation (and therefore bi-allelic expression) of TAL1. We first tested whether bi-allelic TAL1 cases were associated with a general decrease in the expression levels of one or several of the main (co-)factors of PRC2 complex (EZH2, SUZ12, EED, AEBP2). Although a large individual variability in expression levels was apparent, no statistically significant differences (Mann-Whitney test) could be observed in EZH2, SUZ12, EED, or AEBP2 expression levels either according to the mono- *vs.* bi-allelic status (A.) or to TAL1 expression levels (B.). This suggested the absence of a unifying mechanism targeting PRC2 expression in bi-allelic TAL1 cases.

To further test whether loss-of-function of some components of the PRC2 complex might sporadically lead to cases of bi-allelic TAL1 activation, we screened for mutations (by direct sequencing) and/or copy number variation (*i.e.* deletion by CGH-Array and quantitative genomic PCR) of EZH2 and SUZ12, two PRC2 complex components in which mutations were previously reported. Mutations in EZH2 and SUZ12 were only observed in bi-allelic cases (3/47 biallelic tested cases *vs* 0/28 monoallelic (C. and Table S2). Large deletions in SUZ12 were also observed in 6/47 bi-allelic and 2/28 monoallelic cases (D. and Table S2). Of note, the latter corresponded to the two largest deletions observed and are therefore likely to include other genes that SUZ12. None of the mono-allelic cases with insertion contained mutations or deletions in EZH2 or SUZ12.

Because we could not exhaustively screen all possible mutations in all (co-)factors of the PRC2 and PRC1 complexes (most of which are actually unknown in human), we cannot evaluate the extent to which direct or indirect PcG loss-of-function alterations might add-up to contribute

globally to TAL1 trans-activation in biallelic cases. Nevertheless, to the extent of this small screen, our data suggest that this may be a relatively infrequent event which cannot account for the large fraction of bi-allelic TAL1 expression.

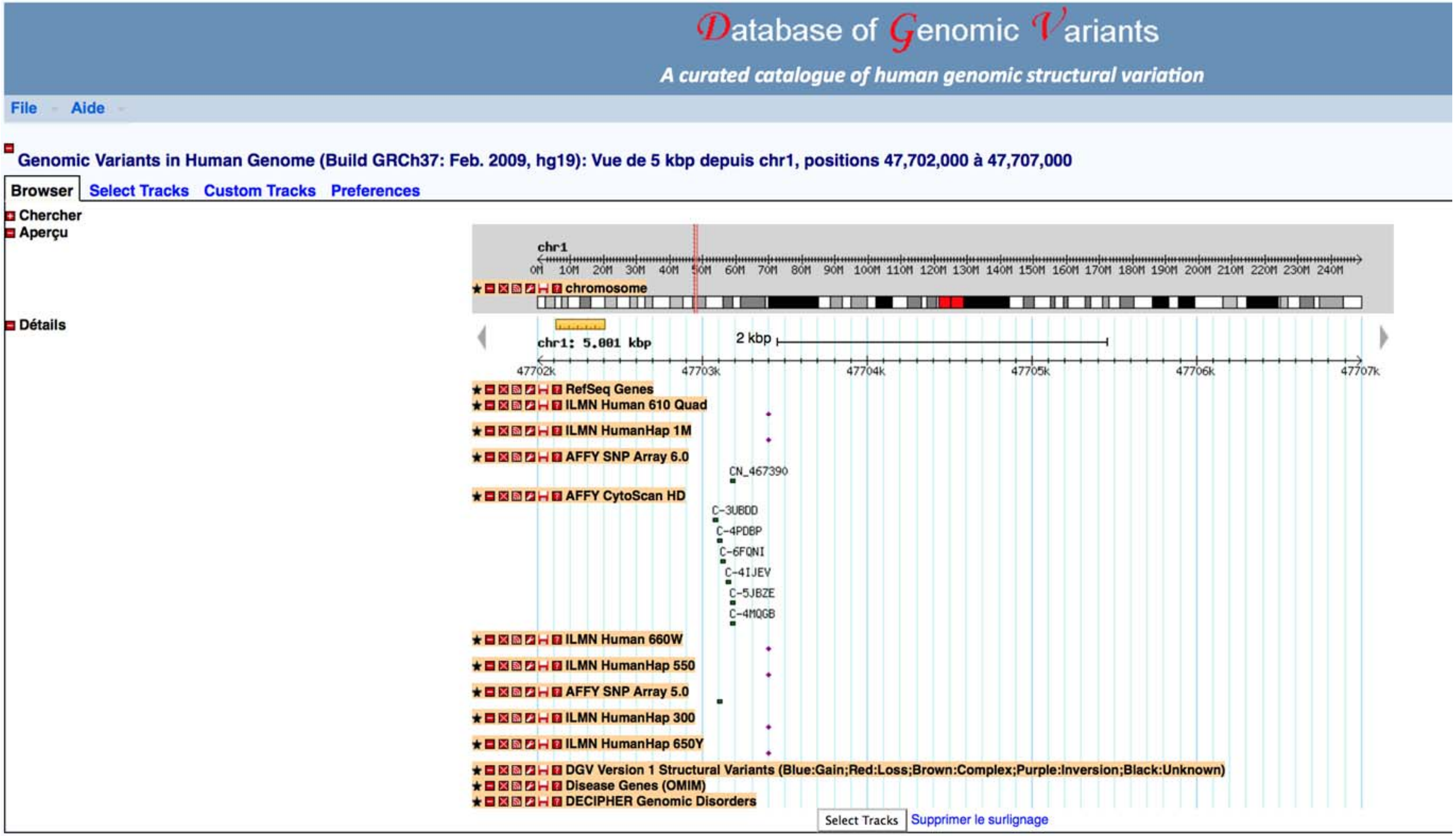
Database of Genomic Variants
A curated catalogue of human genomic structural variation
File
Aide
Genomic Variants in Human Genome (Build GRCh37: Feb. 2009, hg19): Vue de 5 kbp depuis chr1, positions 47,702,000 à 47,707,000
Browser
Select Tracks
Custom Tracks
Preferences
Chercher
Aperçu
Détails
chr1
chromosome
chr1: 5.001 kbp
2 kbp
47702k
47703k
47704k
47705k
47706k
47707k
RefSeq Genes
ILMN Human 610 Quad
ILMN HumanHap 1M
AFFY SNP Array 6.0
CN_467390
AFFY CytoScan HD
C-3UBDD
C-4PDBP
C-6FQNI
C-4IJEV
C-5JBZE
C-4MQGB
ILMN Human 660W
ILMN HumanHap 550
AFFY SNP Array 5.0
ILMN HumanHap 300
ILMN HumanHap 650Y
DGV Version 1 Structural Variants (Blue:Gain;Red:Loss;Brown:Complex;Purple:Inversion;Black:Unknown)
Disease Genes (OMIM)
DECIPHER Genomic Disorders
Select Tracks
Supprimer le surlignage

NCBI Resources How To

dbSNP

SNP | tal1 human

Save search Advanced

Show additional filters

Clear all

Organism
Homo sapiens
More ...

Variation Class
snp

Annotation
nucleotide

Global MAF
Custom range...

Validation Status
by-1000 Genomes
by-frequency

Chromosome Range clear
✓ **From 47234220 to 47239771**

Search fields
Choose ...

Clear all

Show additional filters

Display Settings: Summary, Sorted by SNP_ID

Send to:

Results: 2

Filters activated: from 47234220 to 47239771. Clear all to show 696 items.

1. rs150008850 *[Homo sapiens]*

GAAATTTGGGGGTGCGAAGAAGGGG[G/T]GTGGGGAGAAGCATCAGTATCCAGA

Chromosome:	1:47234227
Gene:	TAL1 (GeneView)
Functional Consequence:	upstream variant 2KB
Validated:	by 1000G,by frequency
Global MAF:	T=0.0068/34
HGVS:	NC_000001.10:g.47699899G>T, NC_000001.11:g.47234227G>T, NM_001290403.1:c.-2218C>A, NM_001290406.1:c.-2092C>A, NM_003189.5:c.-2336C>A, XM_005271158.1:c.-2218C>A, XM_005271158.2:c.-2218C>A, XM_006710863.1:c.-2248C>A

2. rs187837992 *[Homo sapiens]*

GGCTAGAACAGAAGGGTGGTAGGTT[C/G]GTGAAAAGAATAGAAAAGTGTCCAG

Chromosome:	1:47234314
Gene:	TAL1 (GeneView)
Functional Consequence:	upstream variant 2KB
Validated:	by 1000G,by frequency
Global MAF:	G=0.0022/11
HGVS:	NC_000001.10:g.47699986C>G, NC_000001.11:g.47234314C>G, NM_001290403.1:c.-2305G>C, NM_001290406.1:c.-2179G>C, NM_003189.5:c.-2423G>C

Supplementary Figure 8: SNPs in the 5' TAL1 region. No SNPs are referenced at the insertion position Chr1:47,704,964 (HG19) in the Database of Genomic Variants (http://dgv.tcag.ca/dgv/app/home , HG19 coordinates, search chr1:47702000-47707000) or NCBI (http://www.ncbi.nlm.nih.gov/snp , HG18 coordinates, search human tal1, apply chromosome range from 47,234,220 to 47,239,771).

Supplementary Table 1: Clinical features of T-ALL patients

	total	biallelic TAL1	monoallelic TAL1 (without SIL-TAL1 cases)	p-value (χ²)† biall vs mono TAL1 (SIL-TAL1 negative)	biallelic TAL1	monoallelic TAL1 (with SIL-TAL1 cases)	p-value (χ²)† biall vs mono TAL1 (with SIL-TAL1 cases)
	n (%)	n (%)	n (%)		n (%)	n (%)	
	105	69 (66)	20 (19)		69 (66)	36 (34)	
EGIL							
1-2	33/95 (35)	24/62 (39)	5/18 (28)	0.57	24/62 (39)	9/33 (27)	0.37
3	44/95 (46)	27/62 (44)	9/18 (50)	0.79	27/62 (44)	17/33 (52)	0.52
4	18/95 (19)	11/62 (18)	4/18 (22)	0.73	11/62 (18)	7/33 (21)	0.78
Genotype subsets analysed							
CALM-AF10	5 (5)	5 (7)	0 (0)	0.58	5 (7)	0 (0)	0.16
TLX1	18 (17)	16 (23)	1 (5)	0.1	16 (23)	1 (3)	**0.006****
TLX3	11 (10)	9 (13)	1 (5)	0.4	9 (13)	1 (3)	0.1
None of above	76 (72)	39 (57)	18 (90)	**0.007****	39 (57)	34 (94)	**<0.0001*****
Clinical subsets analysed							
Age median	30.5	31.9	32.4	0.76	31.9	27.1	0.09
WBC median	40.11	17.5	81.27	**0.0012****	17.5	112.4	**<0.0001*****
CNS involvement	10 (10)	4 (6)	4 (20)	0.07	4 (6)	6 (17)	0.09
corticosteroide response	51/104 (49)	36/68 (53)	9 (45)	0.61	36/68 (53)	15 (42)	0.31
Complete Response	97 (92)	63 (91)	20 (100)	0.33	63 (91)	34 (94)	0.71

WBC, White Blood Cells; CNS, Central Nervous System

† Patient characteristics were statistically compared by applying Fisher's exact test for categorical variables and the Mann-Whitney test for continuous variables.

Supplementary Table 2: SUZ12 and EZH2 mutations/deletions

SUZ12

		deletion				mutation
UPN patient	*TAL1* allelic expression	chromosomic position	genomic position	size (Mb)	ratio genomic quantitative PCR	
10239	biall	**del(17)(q11.2)**	29 000 018-30 400 871	1.40	0.52	GL
8352	biall	**del(17)(q11.2)**	29 054 354-30 326 121	1.27	0.59	GL
9536	biall	**del(17)(q11.2)**	28 572 030-30 542 594	1.97	0.57	GL
9966	biall	**del(17)(q11.2)**	28 662 829-30 564 494	1.90	0.53	GL
13332	biall	ND	-		**0.56**	GL
9266	biall	**del(17)(q11.2)**	29 601 295-31 044 736	1.44	ND	GL
8941	biall	GL	-	-	ND	**D585fs**
9444	mono	**del(17)(q11.2)**	28 952 285-36 846 112	7.89	0.48	GL
5832	mono	**del(17)(q11.2)**	28 484 282-31 928 577	3.44	0.58	GL

EZH2

		deletion				mutation
UPN patient	*TAL1* allelic expression	chromosomic position	genomic position	size (Mb)	ratio genomic quantitative PCR	
10568	biall	ND	-	-	1.02	**C548fs**
7568	biall	neg	-	-	ND	**Y733X**

Supplementary References

1 Zhang, J. A., Mortazavi, A., Williams, B. A., Wold, B. J. & Rothenberg, E. V. Dynamic transformations of genome-wide epigenetic marking and transcriptional control establish T cell identity. *Cell* **149**, 467-482, (2012).

2 Brandt, V. L. & Roth, D. B. V(D)J recombination: how to tame a transposase. *Immunol.Rev.* **200**, 249-260 (2004).

3 Vanura, K. *et al.* In vivo reinsertion of excised episomes by the V(D)J recombinase: A potential threat to genomic stability. *Plos Biology* **5**, 515-530 (2007).

4 Marculescu, R. *et al.* Distinct t(7;9)(q34;q32) breakpoints in healthy individuals and individuals with T-ALL. *Nat.Genet.* **33**, 342-344 (2003).

5 Merelli, I. *et al.* RSSsite: a reference database and prediction tool for the identification of cryptic Recombination Signal Sequences in human and murine genomes. *Nucleic Acids Res* **38**, W262-267, (2010).

6 Marculescu, R., Le, T., Simon, P., Jaeger, U. & Nadel, B. V(D)J-mediated translocations in lymphoid neoplasms: a functional assessment of genomic instability by cryptic sites. *The Journal of Experimental Medicine* **195**, 85-98 (2002).

7 Hiom, K., Melek, M. & Gellert, M. DNA transposition by the RAG1 and RAG2 proteins: a possible source of oncogenic translocations. *Cell* **94**, 463-470 (1998).

8 Agrawal, A., Eastman, Q. M. & Schatz, D. G. Transposition mediated by RAG1 and RAG2 and its implications for the evolution of the immune system. *Nature* **394**, 744-751 (1998).

9 Marculescu, R. *et al.* Recombinase, chromosomal translocations and lymphoid neoplasia: Targeting mistakes and repair failures. *DNA Repair (Amst)* **5**, 1246-1258 (2006).

10 Marculescu, R. *et al.* Alternative end-joining in follicular lymphomas' t(14;18) translocation. *Leukemia* **16**, 120-126 (2002).

11 Reddy, Y. V. R., Perkins, E. J. & Ramsden, D. A. Genomic instability due to V(D)J recombination-associated transposition. *Genes Dev.* **20**, 1575-1582 (2006).

12 Curry, J. D. *et al.* Chromosomal reinsertion of broken RSS ends during T cell development. *Journal of Experimental Medicine* **204**, 2293-2303 (2007).
